# Developing Components of an Integrated mHealth Dietary Intervention for Mexican Immigrant Farmworkers: Feasibility Usability Study of a Food Photography Protocol for Dietary Assessment

**DOI:** 10.2196/54664

**Published:** 2024-12-13

**Authors:** Isabel Diana Fernandez, Yu-Ching Yang, Wonkyung Chang, Amber Kautz, Karen Farchaus Stein

**Affiliations:** 1 Department of Public Health Sciences, Division of Epidemiology University of Rochester School of Medicine and Dentistry Rochester, NY United States; 2 School of Nursing University of Rochester Medical Center Rochester, NY United States

**Keywords:** Mexican immigrant farmworker, diet-related noncommunicable diseases, mHealth, dietary assessment, image-based, healthcare disparities, minority, feasibility study, food photography, rural health, health literacy, culutural adaptation, women, technology acceptance, mobile health

## Abstract

**Background:**

Rural-urban disparities in access to health services and the burden of diet-related noncommunicable diseases are exacerbated among Mexican immigrant farmworkers due to work demands, social and geographical isolation, literacy issues, and limited access to culturally and language-competent health services. Although mobile health (mHealth) tools have the potential to overcome structural barriers to health services access, efficacious mHealth interventions to promote healthy eating have not considered issues of low literacy and health literacy, and food preferences and norms in the Mexican immigrant farmworker population. To address this critical gap, we conducted a series of preliminary studies among Mexican immigrant farmworkers with the long-term goal of developing a culture- and literacy-specific smartphone app integrating dietary assessment through food photography, diet analyses, and a non–text-based dietary intervention.

**Objective:**

This study aimed to report adherence and reactivity to a 14-day food photography dietary assessment protocol, in which Mexican immigrant farmworker women were instructed to take photos of all foods and beverages consumed.

**Methods:**

We developed a secure mobile app with an intuitive graphical user interface to collect food images. Adult Mexican immigrant farmworker women were recruited and oriented to the photography protocol. Adherence and reactivity were examined by calculating the mean number of food photos per day over time, differences between the first and second week, and differences between weekdays and weekends. The type of foods and meals photographed were compared with reported intake in three 24-hour dietary recalls.

**Results:**

In total, 16 Mexican farmworker women took a total of 1475 photos in 14 days, with a mean of 6.6 (SD 2.3) photos per day per participant. On average, participants took 1 fewer photo per day in week 2 compared with week 1 (mean 7.1, SD 2.5 in week 1 vs mean 6.1, SD 2.6 in week 2; *P*=.03), and there was a decrease of 0.6 photos on weekdays versus weekends (mean 6.4, SD 2.5 on weekdays vs mean 7, SD 2.7 on weekends; *P*=.50). Of individual food items, 71% (352/495) of foods in the photos matched foods in the recalls. Of all missing food items (n=138) and meals (n=36) in the photos, beverages (74/138, 54%), tortillas (15/138, 11%), snacks 16/36, 44%), and dinners (10/36, 28%) were the most frequently missed. Most of the meals not photographed (27/36, 75%) were in the second week of the protocol.

**Conclusions:**

Dietary assessment through food photography is feasible among Mexican immigrant farmworker women. For future protocols, substantive adjustments will be introduced to reduce the frequency of missing foods and meals. Our preliminary studies are a step in the right direction to extend the benefits of mHealth technologies to a hard-to-reach group and contribute to the prevention and control of diet-related noncommunicable diseases.

## Introduction

Mexican immigrant farmworkers are vital to the US agricultural industry [1], yet they and their families are among the poorest, most isolated, marginalized, and underserved groups in the United States [2,3]. While rural Americans face inequities in access to health care resources [4] and burden of diet-related noncommunicable diseases (DR-NCDs) [5,6], those gaps are deepened when considering Mexican immigrant farmworkers as a distinct rural population [7,8]. Mexican immigrant farmworkers have high levels of poverty [2,9] and food insecurity [10] while experiencing long and unpredictable work hours at physically risky and demanding jobs [11,12]. Mexican immigrant farmworkers often live and work in socially and geographically isolated conditions with scarce access to transportation [13], lack of health insurance, and limited access to culturally and language-competent health services [4] and to health-related safety net programs [8,14,15]. In addition, first-generation Mexican immigrant farmworker families describe changes in their dietary intake, reflecting acculturation to the US dietary pattern [16-20]. Within this context, we would expect Mexican immigrant farmworkers to experience disproportionate rates of DR-NCDs. However, the quantification of disparities is elusive due to inconsistent documentation on the health status among Mexican immigrant farmworkers, which is often limited to selected or regional samples and heterogeneous in terms of country of origin and farmworker status. Estimates of the prevalence of diabetes, hypertension, and overweight and obesity are 11%, 14%, and 32%, respectively [21], in a sample of farmworkers (31% children) with access to federally funded Migrant Health Centers. In contrast, regional and local estimates report that among adult Mexican immigrant farmworkers, the prevalence of diabetes is 17% and prediabetes is 60% [22], hypertension is between 55% and 69% [22,23], and overweight and obesity is between 64% and 92% [22,23]. Since dietary intake is among the key modifiable factors that contribute to DR-NCDs [24], efficacious interventions to promote healthy eating and overcome structural barriers to health care access (particularly health-promoting care) in this population will contribute to the primary and secondary prevention of DR-NCDs.

Culturally adapted mobile health (mHealth) tools that take into account Mexican immigrant farmworkers’ unique constellation of personal (eg, cultural meanings and level of health literacy), economic (eg, food insecurity), and environmental (eg, limited transportation and long work hours) constraints to improve access to health services and reduce health inequities have been suggested [25,26]. While access to weight control and healthy eating interventions have improved as the internet and cell phones have become a common mode of delivery, these interventions have been most effective in persons of high socioeconomic status [27], have not considered the issue of low literacy and low health literacy [28], and have failed to address cultural food preferences and eating norms [29]. Although there is evidence of high rates of cell phone ownership and willingness to use mHealth devices among Mexican immigrant farmworkers [30,31], the unavailability of mHealth dietary intervention for Mexican immigrant farmworkers has become another source of increasing disparities. For example, self-monitoring of dietary intake, one commonly used mobile app intervention [32], requires detailed documentation of food intake using complex word-based programs that generally do not include ethnic-specific food choices.

Further complicating access to effective mHealth diet interventions in Mexican immigrant farmworkers is the need for a reliable dietary assessment, a critical first step to the delivery of tailored dietary interventions. Currently, self-reported dietary assessment methods require literacy (eg, food records), the ability to average out frequency of eating over time (eg, food frequency questionnaires), or a bilingual-bicultural interviewer (eg, 24-hour dietary recall) [33]. Diet assessment through food photography, although still in its infancy, has the potential to overcome these limitations [34]. The development of a food image recognition diet assessment tool linked with a non–text-based, culturally adapted, healthy eating intervention as a smartphone app is a highly innovative step toward the delivery of nutritional care to underserved, difficult-to-reach populations and to the prevention and control of diet-related health inequities.

To address this critical gap, we conducted a series of preliminary studies among Mexican immigrant farmworkers to test the feasibility of collecting dietary intake data through a smartphone camera app and to develop a working draft of a preventive dietary intervention that integrates behavior change strategies with cultural (Mexican food traditions and preferences) and socioeconomic factors (eg, work demands and limited transportation to grocery stores) influencing their intake patterns. Our long-term goal is to leverage mHealth technologies to reduce the gaps in access to nutrition-preventative services and contribute to the primary and secondary prevention of DR-NCDs by developing a culture- and literacy-specific smartphone app with 3 integrated components: dietary assessment through food photography, analyses of dietary intake to identify problem intake, and a tailored and culturally relevant dietary intervention that is accessible across varying levels of literacy and health literacy. Here, we are reporting on the feasibility of using a smartphone camera app to assess long-term dietary intake in a sample of farmworker women of Mexican origin. Specifically, we examined adherence and reactivity to a food photography protocol and characterized adherence by type of foods and eating occasions by comparing the content of the food photos with a validated dietary assessment method. We focused on women because of their worries about body weight and related diseases [16] and their central role in the procurement and preparation of food for themselves and their families [35-37].

## Methods

### Study Overview

The study was conducted in farming communities in Western New York from April 2017 to August 2018. The study consisted of a 14-day food photography period during which participants were asked to take photos of all food and beverages consumed using a camera app on a project-loaned Android (Google) smartphone. Fourteen days of food photographs including 2 weekends allowed us to examine differences in adherence between weekdays and weekends. It also enabled the examination of reactivity to the food photography protocol, defined as changes in adherence caused by the recording process [38] to identify the number of days needed to obtain details on dietary patterns while minimizing participant burden. Three 24-hour dietary recalls (24H-DRs), a validated dietary assessment tool [33,39,40], were administered during the food photography period to further characterize protocol adherence by identifying the missing foods and eating occasions (eg, dinners). These observations will inform the design of the final food photography app.

### Participant Eligibility and Recruitment

Study participants were women who met the following eligibility criteria: (1) self-identified as being of Mexican origin; (2) aged 21 to 45 years, purposively targeting an age group typically balancing outside the home and multiple family responsibilities; and (3) living in a farming community in Western New York. Women were ineligible if they were pregnant or lactating, or were following a medically prescribed diet (eg, diabetic diet and weight loss diet).

Women were recruited by native Spanish-speaking, bilingual data collectors who had established relationships with the regional farmworker communities. The data collectors were well-known in the communities through employment-related outreach and advocacy. Study participants were recruited based on the data collector’s knowledge of the community, a modified snowball recruitment technique [41], and referrals from cooperating community-based organizations and community leaders. Data collectors contacted women by phone or in person to inform them of the study; determine their interest in participating; and for those interested, complete a brief screening interview. To reduce the risk of coercion, 2 additional visits were offered. For women eligible and interested in participating, a second meeting was scheduled to discuss the study, review the informed consent, and answer questions. For those who preferred to discuss participation with friends and family, a copy of the consent was provided and a third visit to complete the consent was offered. Women interested in participating completed the process of informed consent.

### Study Procedures

The study protocol consisted of a total of 5 face-to-face data collection visits at the participant’s home or a location of their choice (eg, a private room in a public library). All written materials were prepared at a sixth-grade reading level and were read aloud by the data collector. The orientation visit took approximately 1 hour and included hands-on instruction on the overall use of the phone, how to use the camera app, and how to photograph foods and beverages. Participants were asked to always carry the cell phone with them to take photos of all foods and beverages in all meals (including snacks) before consumption and to avoid capturing human faces in the photos. After completing the orientation, questionnaires were administered verbally and participants had weight and height measured to calculate BMI (weight in kg/height in m^2^).

In total, 3 face-to-face visits occurred during the 14-day food photography period. The meetings were scheduled based on the participants’ availability and were approximately equally distributed across the 14-day period (eg, days 3, 7, and 10). During these visits, the data collector began by reviewing the quality of food photographs with participants. Images that were duplicates, were nonfood relevant, or included faces or other identifiable information were removed. At each face-to-face visit, participants were administered a 24H-DR (a total of 3) by a trained diet technician using the Nutrition Data System for Research (NDSR) software and multiple pass methodology (version 2016) developed by the Nutrition Coordinating Center, University of Minnesota, Minneapolis [42]. The data collector served as a translator and assisted with portion size estimation using common kitchen measuring tools. The final face-to-face visit occurred within 1 week of completing the food photograph protocol. During that session, new photos were reviewed, the incentive for participation was paid, and the project smartphone was collected.

### Data Collectors and Training Activities

Training of the data collector consisted of approximately 46 hours of activities including (1) Collaborative Institutional Training Initiative human participants program; (2) university-specific training; (3) project-specific instruction on participant recruitment, informed consent processes, data collection, and management; (4) role-playing; and (5) observation in the community of participant enrollment and data collection sessions. All training was done by the project manager and the principal investigator.

After 9 participants were recruited, irregularities in processes and questionnaire responses (eg, identical responses for all participants) were identified during routine monitoring. Initial efforts to address the problems were unsuccessful. Therefore, enrollment and data collection activities were stopped at month 2 of the project and additional training for the data collector was provided. Shortly thereafter, the data collector resigned. A second data collector was hired and trained using the same processes described above. Data collection resumed in December 2017.

### Measures to Characterize the Sample

Demographic questionnaire included standard items (eg, age, educational level, and country of origin). Weight and height were measured with participants wearing light clothing and in privacy with a portable autocalibrated digital scale to the nearest 0.1 kg (EatSmart model ESBS-03) and Shorr Infant/Child/Adult Height/Length measuring board stadiometer to the 0.1 cm, respectively. Measures were used to calculate the BMI as weight/height in kg/m^2^.

The Acculturation Rating Scale for Mexican-Americans II (ARSMA-II) [43] measured bicultural acculturation with 2 orthogonal subscales, the Mexican Orientation Scale (17 items) and the Angelo Orientation Scale (13 items), with evidence of its reliability and validity with Spanish-speaking Mexican adults. The measure is behaviorally focused and addresses spoken language and identification in everyday activities. Items are rated on a 5-point scale anchored by “not at all” and “almost always.” The scale scores are computed by averaging across items.

The Newest Vital Signs instrument [44] addressed reading and numeracy aspects of health literacy particularly related to food labels and nutrition, with evidence of its reliability and validity. Participants were presented with a nutrition label from a container of ice cream and asked to answer 6 questions to test their ability to read basic text and perform simple mathematical computations. Furthermore, 1 point is assigned with each correct response with a score of 0-1 indicating high likelihood of limited literacy, a score of 2-3 indicating a possibility of limited literacy, and a score of 4-6 indicating adequate health literacy.

The 24H-DR is a validated tool to measure self-reported food and beverage intake over the previous 24-hour period that does not require respondent’s literacy [33,39,40]. Participants are asked to recall the time of intake, type of meal (eg, dinner), type of food and beverages, and the portion size. The 24H-DR served two purposes: (1) to characterize adherence to the food photography protocol by comparing the type and timing of food items and eating occasions captured by the photos against the recalls and (2) to describe participants’ dietary intake by analyzing the data using NDSR software version 2016 [42].

### Mobile Food Photography App

An Android mobile app was developed by our collaborators in computer science to collect food images. The password-protected app uses the built-in camera and provides an intuitive graphical user interface to display simple prompts and take and review pictures. The app resembled a typical camera app but was redesigned for ease of use and the protection of food imaging by storing them in a separate location from the standard cell phone photo folder. Photos were automatically identified by participant ID number, date, time, and photo ID number only and stored in the cell phone password-protected gallery, separate from the typical photo gallery, accessible only to the data collector during the 14-day recording period. After completion of data collection, the photo images were downloaded to secure servers. Photos were then examined for quality (deleted blurred photos), and duplicates (eg, 2-3 photos of noodle soup based on the time stamp) and nonfood photos (eg, photo with a pair of glasses) were deleted.

To enhance photo protocol adherence, 2 features were built into the app. One was a daily summary SMS text message automatically sent from the participant’s project cell phone to the project staff member detailing the number and timing of photos taken each day. If changes in the pattern of photos were noticed, a phone call from the data collector followed. The other feature consisted of 3 SMS text message reminders per day sent at fixed times encouraging participants to take photos of all food and beverages.

### Data Analysis and Management

Questionnaires were checked for completeness and manually entered into REDCap (Research Electronic Data Capture; Vanderbilt University) by a study staff member and later checked by another study member for accuracy. The distributional properties and the presence of outliers were examined for each variable. A research team member did a final review of the photo dataset to remove duplicates (based on the time stamp), blurred photos, and photos containing no food items (eg, a photo with a pair of glasses).

We described the demographic characteristics and the BMI of the sample (means, medians, ranges, and proportions) and computed scale scores for the health literacy and acculturation measures (New Vital Signs and ARSMA, respectively). In addition, dietary data from the three 24H-DRs were averaged to estimate the number of servings of fruits and vegetables (in cups equivalent), the proportion of grains as whole grains, and the percentage of total calories from sugar added and saturated fats. We contrasted those dietary intake estimates to current dietary recommendations [45].

We did not establish an a priori definition of adherence to the food photography protocol because the daily meal patterns of our Mexican immigrant farmworkers were previously unknown and understanding them was part of our investigation. To answer whether farmworker women adhere to a 14-day food photography protocol and if there were signs of reactivity to it, we computed the mean number of photos taken each protocol day and compared the mean number of photos between week 1 and week 2, and the mean number of photos between weekday and weekend days with a 2-tailed *t* test (*P*<.05) among all participants. We additionally plot the mean number of photos in a given hour of the day, starting at midnight and ending at 11 PM, averaged for each person across the 14 days and then averaged among all participants to identify general daily mealtime patterns (no formal test performed).

To determine the completeness of the food photographs content, we compared the number and type of food and beverage items (herein food items) between the 24H-DR (the reference) and the food items in the photo images taken over the same 24-hour period (3 days of food photography per participant compared with the same 3 days recalled in the 24H-DR). For this study, a food item was defined as a distinct food, independent of the ingredients involved (ie, a bowl with rice, beans, and cheese equals 1 food item; a plate with a fried egg and French fries equals 2 items). Two of the coauthors (KY and AK) independently matched the number and type of food items between the photos and 24H-DR (the reference) using the time and date stamped in the photos and the 24H-DR time and date of food intake recalled; they later met to compare results. Any disagreements were resolved at a team meeting by consulting a senior researcher (IDF). We first computed the percentage of the total number of food items documented in 3 days of photographs out of the total number of food items in the concurrent three 24H-DRs (total number of food items in photographs/total number of food items in the recalls). Second, we calculated the percentage of food items in the photos that match in type of food and time of intake to the food items in the recall (number of food items in the photos matched to food items in the recalls/total number of food items in the recalls). Unmatched food items were due to missing food items in the photos or food items that matched in time of intake but differed in the type of food item. We examined the frequency and characteristics of unmatched food items and meals (breakfast, lunch, dinner, and snacks) from the photos (eg, participant recalled roasted chicken at dinner time that was not photographed).

### Ethical Considerations

This study was approved by the University of Rochester Research Subjects Review Board (study ID 00001150). Due to risks for undocumented participants, consent was verbal and witnessed by the data collector’s signature. No identifying information was collected. Participants received an incentive for their participation (a total of US $77).

## Results

### Overview

A total of 25 women were enrolled in the study. However, data from the first 9 participants (obtained by the first data collector) were not included in our analyses due to missing all demographics except for age and birthplace, and questionable quality of surveys (eg, all participants had the same responses). Consequently, the analytic sample for this study is 16 women. Compared with the analytical sample, the excluded participants did not differ in median age (nonparametric test *P* value=.08), and 6 (66%) of the 9 were born in the United States (chi-square test *P*<.001). Participants were early- and middle-aged women, all of whom were born in Mexico. Most participants had either overweight or obesity (15/16, 94%), had less than a high-school diploma (15/16, 94%), retained a Mexican cultural orientation (15/16, 94%), and were of limited or possible literacy (11/16, 69%). Both the age at immigration to and the length of stay in the United States varied considerably across the sample (Table 1). All cell phones were returned. No participant dropped out of the study.

**Table 1:** Characteristics of participants.

Characteristic		Overall (n=16)
**Age (years), mean (range^a^)**		37.1 (21-45)
**Birthplace (Mexico), n (%)**		16 (100)
**Age at immigration (years), mean (range)**		24.5 (3-37)
**Years in the United States, mean (range)**		13.5 (2-22)
**BMI (kg/m^2^), median (IQR^b^; range)**		30.2 (26.9-33.7; 24.2-41.3)
**Weight, n (%)**		
	Healthy weight (BMI 18.5 to <25 kg/m^2^)	1 (6)
	Overweight (BMI 25 to <30 kg/m^2^)	7 (44)
	Obese (BMI ≥30 kg/m^2^)	8 (50)
**Education level, n (%)**		
	Sixth grade or below	6 (38)
	Middle school	6 (38)
	Some high school	3 (19)
	Graduated high school	1 (6)
**Work type, n (%)**		
	Farming or food processing	16 (100)
	Other	0 (0)
**ARSMA-II^c^, n (%)**		
	Very Mexican oriented	13 (81)
	Mexican oriented to balanced bicultural	2 (13)
	Slightly Anglo-American to oriented bicultural	0 (0)
	Very Anglo oriented	1 (6)
**New vital signs, n (%)**		
	Limited literacy	2 (13)
	Possible literacy	9 (56)
	Adequate literacy	5 (31)

^a^Range: minimum-maximum.

^b^IQR: 25th-75th percentile.

^c^ARSMA-II: Acculturation Rating Scale for Mexican-Americans II.

### Dietary Intake by 24H-DR

In total, 2 recalls were excluded from analyses due to implausible values (daily caloric intake <500 and >3000) [46]. Compared with the 2020-2025 Dietary Guidelines for Americans [45] (Table 2), more than 60% of our participants exceeded the guidelines for percentage of total calories from saturated fats (10/16, 62%) and added sugars (11/16, 69%) and were below recommendations for percent of all grains as whole grains (10/16, 62%). None reached the recommendations for fruits and vegetables intake based on a 2000 calories/day diet for women with moderate physical activity [45].

**Table 2:** Dietary intake^a^ (n=16). Comparison with 2020-2025 Dietary Guidelines for Americans.

Category	Dietary intake	Dietary Guidelines for Americans 2020-2025 [45]
Percent total calories from saturated fat, median (IQR^b^; range^c^)	10.7 (9.6-11.7; 5.8-13.8)	<10%
Percent total calories from added sugars, median (IQR; range)	12.6 (9.4-14.8; 5.4-26.5)	<10%
Percent of grains as whole grains, median (IQR; range)	39.9 (32.8-57.1; 10.0-87.1)	≥50%
Fruits and vegetables, cup equivalents (IQR; range)	2.0 (1.1-2.7; 0.5-3.6)	4.5^d^

^a^Average of three 24-H DR.

^b^IQR: 25th-75th percentile.

^c^Range: minimum-maximum.

^d^Based on a 2000 calories/day diet (caloric requirement for moderate physical activity for women aged 21-25 years is 2200 and aged 26-45 years is 2000).

### Adherence to Food Photography Protocol

A total of 1475 photos were taken by 16 participants over the 14-day food photography protocol (minimum of 63 total on day 14 and maximum of 131 total on day 9). Each day, 14 participants took at least 1 photo (minimum 1 and maximum 16). Two participants took 0 photos on protocol day 14. The mean daily number of photos participants took per protocol day was 6.6 (SD 2.3) photos/day (median 6.5, IQR 6.1-7.3). There was no decreasing nor increasing monotonic trend in the daily mean number of photos across the 14 days (Multimedia Appendix 1). In the last 5 days of the protocol, however, the mean and median number of photos per day were below the overall mean and median across the 14 days. The last day of the protocol had the poorest adherence (2 participants took 0 photos and 8 participants took 1-4 photos; data not shown). On average, participants took 1 fewer photo per day in week 2 compared with week 1 (week 1: mean 7.1, SD 2.5 vs week 2: mean 6.1, SD 2.6; t_15_=–2.47, *P*=.03), and the differences were minimal between weekdays and weekends (weekdays: mean 6.4, SD 2.5 vs weekends: mean 7, SD 2.7; t_15_=–1.15, *P*=.50; Multimedia Appendix 2). Eating episodes occurred continuously throughout the day, especially between 6 AM and 10 PM, with a clear cluster around noon. No clear evening mealtime was evident across the sample (Multimedia Appendix 3).

### Comparison of the Food Photograph Contents With the 24H-DR

Participants reported a total of 495 food items in the 3 days of 24H-DR and 414 food items in the 3 concurrent days of photos. Using 24H-DR as a reference, 352 food items in the photos matched in time of intake and content with the food items in the recalls (352/495, 71% concordance), 5 matched in time of intake but not in the content (5/495, 1% mismatched items; Table 3), and 138 were missing from the photos (138/495, 28%). Beverages were the food items most frequently missing in the photos (74/138, 54%), primarily water (n=54), juice or soda (n=12), and coffee or tea (n=6). Other missing food items were tortillas (15/138, 11%), fruits (12/138, 8.7%), and snacks (11/138, 8%). In addition, women did not take photos of 36 entire meals; 27 (75%) of them occurred in the second week of the protocol, of which 70% (19/27) were in the last 5 days. Snacks were the meals women missed photographing the most frequently (16/36, 44%), followed by dinners (10/36, 28%), lunches (6/36,17%), and breakfasts (4/36,11%). Incidentally, we found 57 food items in the photos that participants did not report in the recalls, of which the most frequent food items were beverages (13 waters and 8 juices or sodas; total 21/57, 37%), snacks (n=5), and fruits (n=6).

**Table 3:** Mismatched food items.

Photo (n=414 food items)	24H-DR^a^ (n=495 food items)
Tortilla	Donut cake
Chicken pasta	Spaghetti
Chicken soup, rice, and vegetables	Chicken veggies
Crackers	Croissant
Strawberry	Cherries

^a^24H-DR: 24-hour dietary recalls.

## Discussion

### Principal Findings

The data presented in this paper are part of a series of preliminary studies leading to the development of a smartphone app with an integrated dietary assessment tool based on food photography, the capacity to analyze food intake and identify problem intakes, and the delivery of tailored evidence-based strategies for dietary modification adapted to Mexican immigrant farmworker culture and level of literacy. Here, we investigated whether Mexican immigrant farmworkers adhere to a 14-day food photography protocol to capture patterns of dietary intake during weekdays and weekends and detect if reactivity occurred. We found that this sample of 16 young and middle-aged Mexican farmworker women, the majority of which conserved a strong Mexican cultural orientation and had a varied level of reading and numerical literacy, adhered to the food photography protocol, albeit with evidence of change over time and considerable variability at the individual level. Using the 24H-DR as the reference assessment tool, women did not take photos of all the meals and foods reported as consumed in the recalls. Although to a lesser extent, the opposite was also true—women did not report in the recalls some of the foods that had been photographed.

### Comparison With Previous Work

Reports on adherence to image-based dietary assessment protocols in the United States included participants of diverse socioeconomic [47-53] and racial and ethnic backgrounds [50-52] and those who are proficient in English [47-53]; among those reporting education, most participants had a high-school education and above [50-52]. Only 2 studies reported adherence data in a rural setting, 1 in low-income child-mother dyads [52] and the other in adults [53], but none of them represented the particular demographic characteristics and environmental challenges of Mexican immigrant farmworkers. The protocols implemented differed from this study in the length of the dietary assessment period (3-7 days) [47-53], the inclusion of photos of foods before and after consumption [47,49-53], inclusion of text or voice description of foods hard to identify from a photo [49,51,52], the type of meals requested to be photographed (only breakfast [51] and only dinner [52]), and measurement of protocol adherence [49,51-53]. As opposed to previous studies of adherence [48,51], we did not establish an a priori definition because the daily meal patterns of our Mexican immigrant farmworkers were unknown and understanding them was a goal of our investigation. Among studies assessing full day of intake, those reporting number of photos as an adherence measure, had means ranging from 3 [48,50] to 10 [47] photos/day across a 7- and 3-day protocol, respectively. Although our participants’ mean photos/day (6.6 photos) sits in the middle of previous studies, our protocol included only photos before consumption, thus lessening participant’s burden compared with prior work. To our knowledge, no US studies examine reactivity to food photography protocols. In our sample of Mexican immigrant farmworker women, we observed that women took fewer photos during weekdays than weekends, perhaps due to known restrictions on cell phone use in the farms, and during the second week, potentially due to fatigue. Participants did not seem to have a clear pattern of mealtimes. Outside of lunchtime, there were food photos at every hour from 6 AM to 10 PM. From our other preliminary studies, we learned that work breaks and start and end times differ according to the type of activity and season (unpublished data).

To our knowledge, previous pilot studies did not use other methods of dietary assessment to further characterize adherence to the food photography. Most of the food items not captured by the photos were foods consumed casually or during working hours when participants had restrictions to use cell phones (eg, beverages and snacks). Furthermore, most of the meals not photographed were snacks and dinners, possibly indicating their unplanned nature (snacks), after-work fatigue, or competing priorities such as family responsibilities (dinners). Comparable to another pilot study among adults [50], breakfast was rarely missed in the photos, perhaps due to its predictability in time and location. Supporting the presence of second-week reactivity, the overwhelming majority of missed meals were in the second week of the protocol. Unexpectedly, some of the food items photographed were not reported in the recalls. Interestingly, the food items missing in the recall were of similar nature to those missing in the photos: namely, beverages and snacks. The latter may be due to memory [40] of foods eaten casually or photos of foods women photographed but did not finally consume. Finally, the 5 mismatched items we found can be explained either by a faulty translation or an error in data entry in the dietary software. The presence of social desirability or eating behavior changes that can occur with both recalls and food photography cannot be ruled out, but none of them would have had an effect on adherence or reactivity to the protocol.

### Conclusions

Based on these findings, we will make substantive adjustments to our food photography protocol. Participants’ training will include awareness of items and meals frequently missed (eg, water and snacks). To avoid the fatigue observed in the second week, we will shorten the length of the assessment period to a week or less. Automated SMS text message prompts adapting ecological momentary assessment methods [54] will be sent at specific times (eg, dinner time) as reminders to take photos. To record eating occasions forgotten or unable to photograph, we will include an option to voice-record missed meals after the actual eating events occurred instead of in real time [55] and automatic reminders to do so. We expect the shorter assessment period, the automated reminders, and the voice-recording option to reduce the frequency of missing food photos. The study reported here intended to broadly understand if and how Mexican immigrant farmworker women would adhere to food photography for dietary assessment and did not attempt to quantify the actual amount consumed based on food photos. Thus, women were asked to photograph food only before consumption. We will enhance our next protocol by adding best practices for food photos [56], such as the appropriate photo angle and lighting, to take photos of second servings and before and after consumption (plate waste) for the correct quantification of foods and food groups intake.

The dearth of studies on all aspects of image-assisted and image-based dietary assessment including diverse population with limited English proficiency, literacy, and access to health services, such as Mexican immigrant farmworkers, represents a missed opportunity for the use of technologies that have the potential to overcome those barriers. Our preliminary studies leading to the development of a culture- and literacy-specific smartphone app are a step in the right direction. We expect that in the context of the Mexican immigrant farmworker environment, this type of app will contribute to reducing inequities in access to preventive nutrition services and, in the long run, to the prevention and control of DR-NCDs.

## Appendix

Multimedia Appendix 1 Mean number of photos across 14-day protocol (n=16).

Multimedia Appendix 2 Mean total number of photos taken. Comparison week 1 vs week 2 and weekdays vs weekends (n=16).

Multimedia Appendix 3 Mean number of photos per hour across 24 hours (n=16).

## Abbreviations

24H-DR: 24-hour dietary recall
ARSMA-II: Acculturation Rating Scale for Mexican-Americans II
DR-NCD: diet-related noncommunicable disease
mHealth: mobile health
NDSR: Nutrition Data System for Research
REDCap: Research Electronic Data Capture
