# Supplementary figures and images for: Developing Components of an Integrated mHealth Dietary Intervention for Mexican Immigrant Farmworkers: Feasibility Usability Study of a Food Photography Protocol for Dietary Assessment
Source: JMIR Form Res. 2024 Dec 13;8:e54664. doi: 10.2196/54664 (PMC11681282; doi:10.2196/54664)

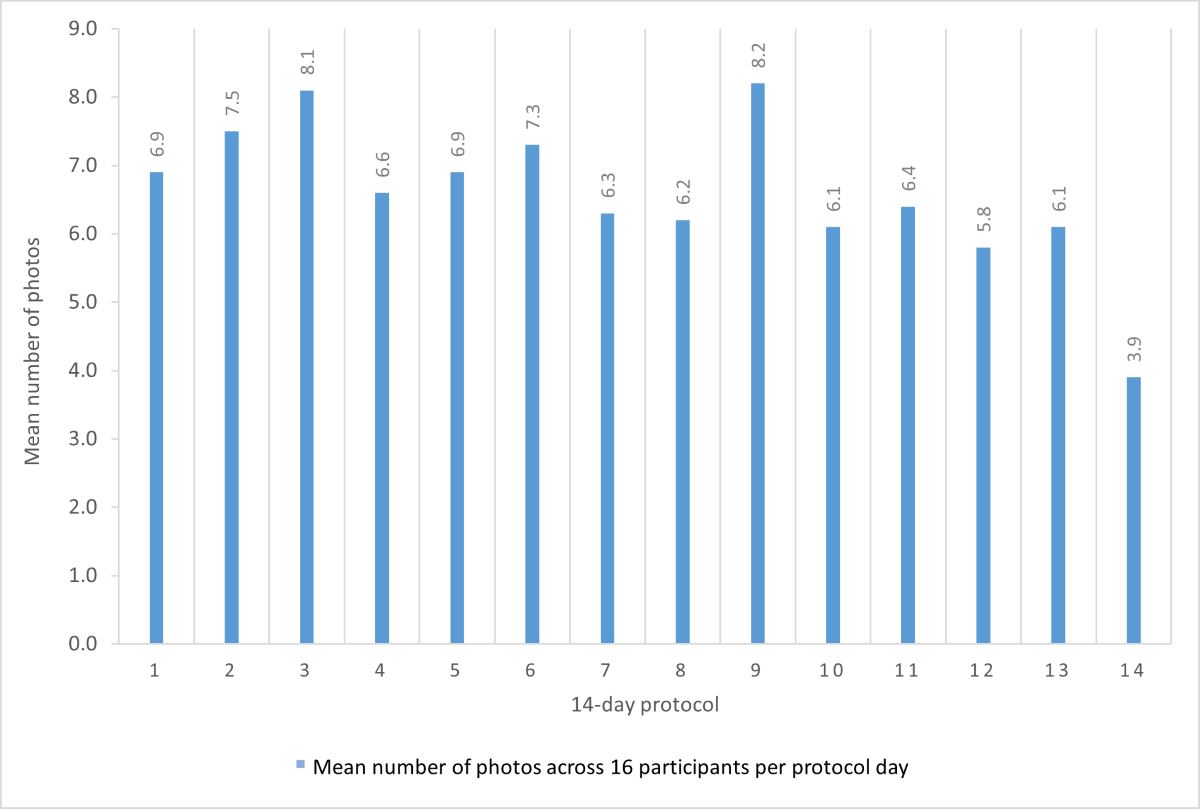

Supplement: Multimedia Appendix 1 [file formative_v8i1e54664_app1.png]

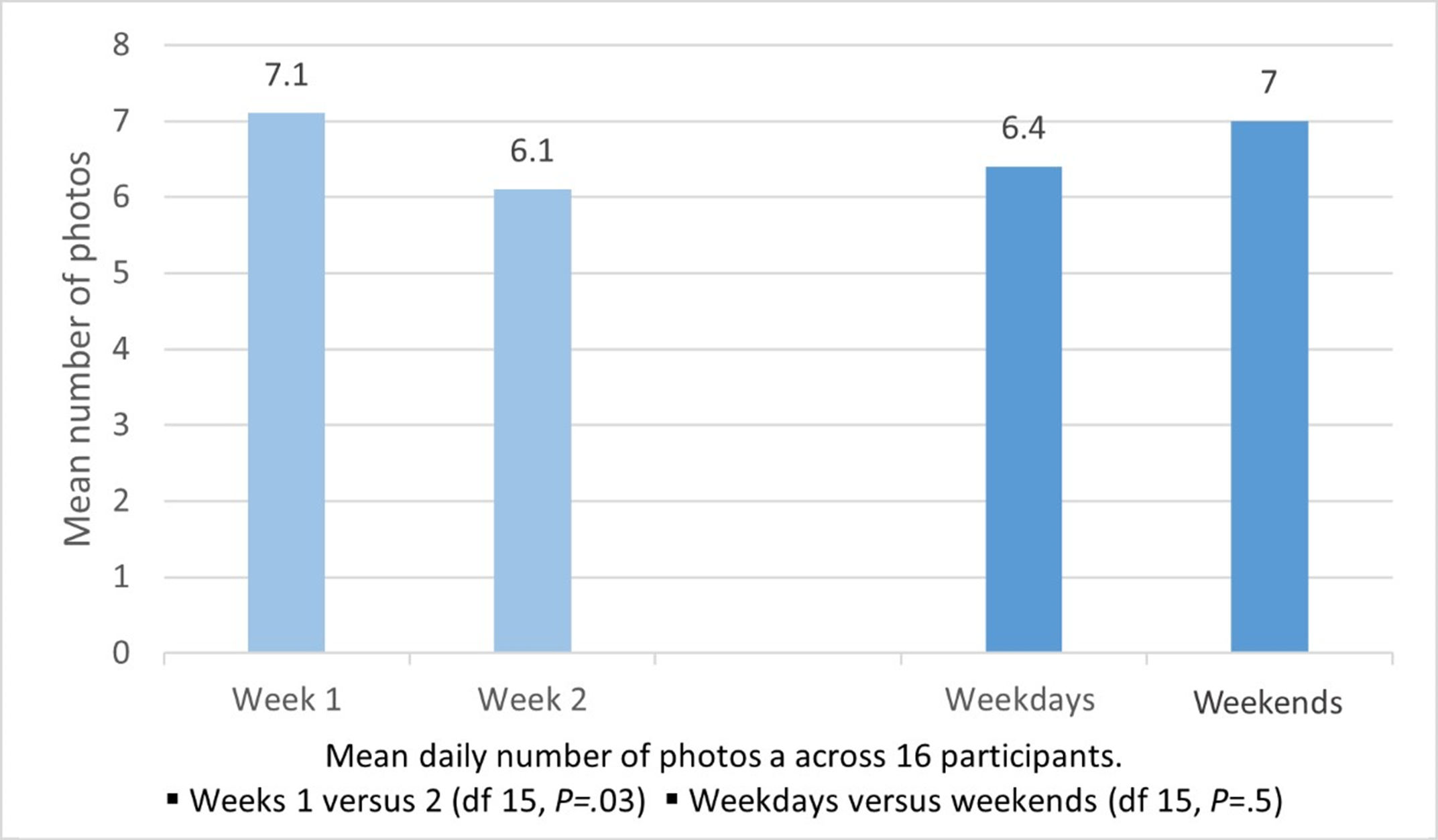

Supplement: Multimedia Appendix 2 [file formative_v8i1e54664_app2.png]

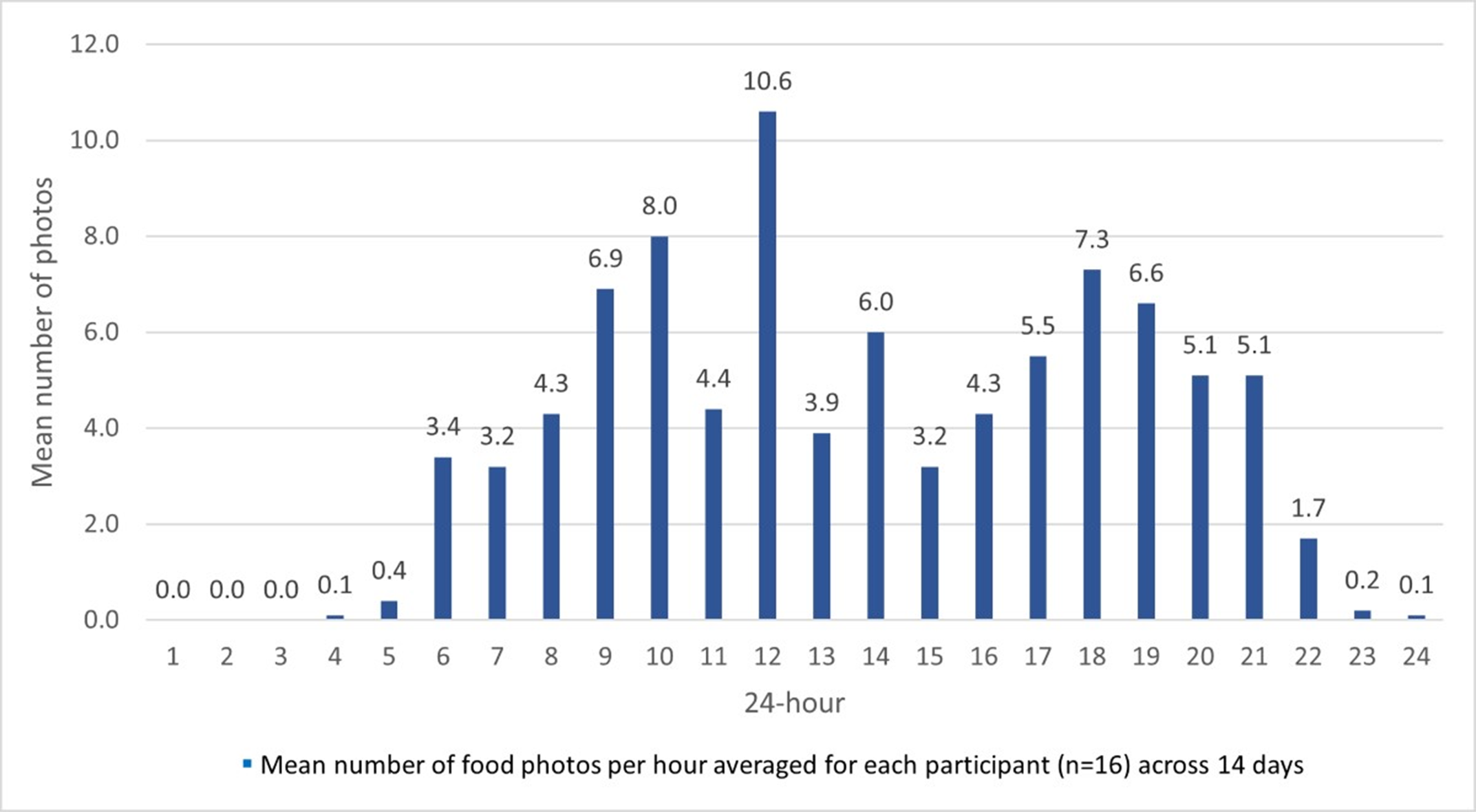

Supplement: Multimedia Appendix 3 [file formative_v8i1e54664_app3.png]
